# Establishing Newborn Screening for SCID in the USA; Experience in California †

**DOI:** 10.3390/ijns7040072

**Published:** 2021-10-31

**Authors:** Jennifer M. Puck, Andrew R. Gennery

**Affiliations:** 1 Department of Pediatrics, School of Medicine, University of California San Francisco, UCSF Benioff Children’s Hospital, San Francisco, CA 94143, USA; 2 Children’s Bone Marrow Transplant Unit, Translational and Clinical Research Institute, Newcastle University, Great North Children’s Hospital, Newcastle upon Tyne NE1 4LP, UK; andrew.gennery@newcastle.ac.uk

**Keywords:** severe combined immunodeficiency (SCID), T-cell receptor excision circle (TREC), primary immunodeficiency, T-cell lymphopenia

## Abstract

Newborn screening for severe combined immunodeficiency (SCID) has developed from the realization that infants affected with SCID require prompt diagnosis and treatment to avoid fatal infectious complications. Screening DNA from infant dried blood spots for T-cell receptor excision circles (TRECs), byproducts of normal antigen-receptor gene rearrangement, has proven to be a reliable method to identify infants with SCID and other serious T lymphocyte defects before the onset of serious infections. The experience of the SCID newborn screening program in California after screening over 3 million infants demonstrates the effectiveness of this measure.

## 1. Introduction to Severe Combined Immunodeficiency (SCID)

Severe combined immunodeficiency (SCID) is a rare lack of adaptive immunity resulting in the inability to fight infections. Affected individuals have no T lymphocytes and absent or dysfunctional B lymphocytes that fail to produce specific antibodies. Therefore, recurrent infections develop, generally by the age of 2–4 months as maternally derived IgG antibodies wane. The infections, which include those caused by opportunistic organisms, fail to resolve and become increasingly severe, such that the disease is not survivable unless patients are supplied with an immune system. At least 18 genes are known that cause SCID [1], and there are also some still unknown. The most commonly found known genes and their distinct T, B, and NK cell profiles are shown below (Table 1).

All SCID genotypes lack naïve T cells, while some have B cells, NK cells, or both. SCID gene products include components of cytokine signaling pathways, the machinery for recombination of the T- and B-cell antigen receptors, or, in the case of adenosine deaminase (ADA) deficiency, purine metabolism.

## 2. Justification for Performing Newborn Screening for SCID

**Figure 1:**
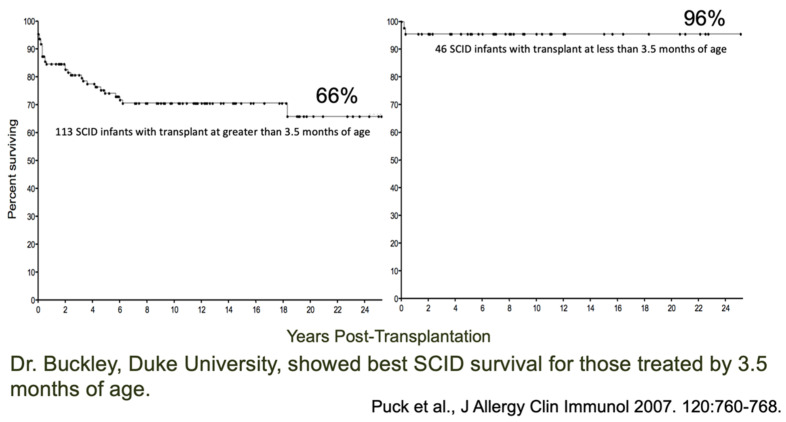
SCID patients treated early have better survival [2].

Below are data that led to the idea that universal newborn screening (NBS) for SCID could be helpful (Figure 1) [2]. Dr. Rebecca Buckley at Duke University noted in her large cohort of patients receiving hematopoietic cell transplants for SCID that the infants transplanted before the age of 3.5 months (right) had far better survival (96%) than those transplanted later (left). The ones on the right were infants who had an affected family member, alerting the family and doctors to the diagnosis right at birth or even prenatally.

**Figure 2:**
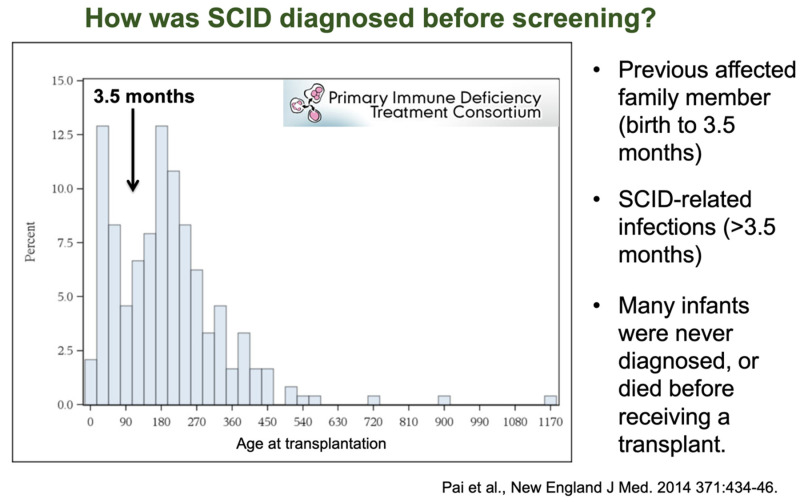
PIDTC SCID longitudinal study data showing a biphasic distribution of age at transplantation [3].

Similar data in the next two illustrations were obtained from the very large multi-center study by the Primary Immune Deficiency Treatment Consortium (PIDTC) [3]. The biphasic age of transplant among SCID patients treated between 2000 and 2010 reflected a smaller, younger group, detected and transplanted before 3.5 months of age due to having a positive family history; and a larger group diagnosed only after developing infections. We assume that many additional infants with sporadic SCID were never diagnosed during this time and died before being able to receive a transplant (Figure 2).

**Figure 3:**
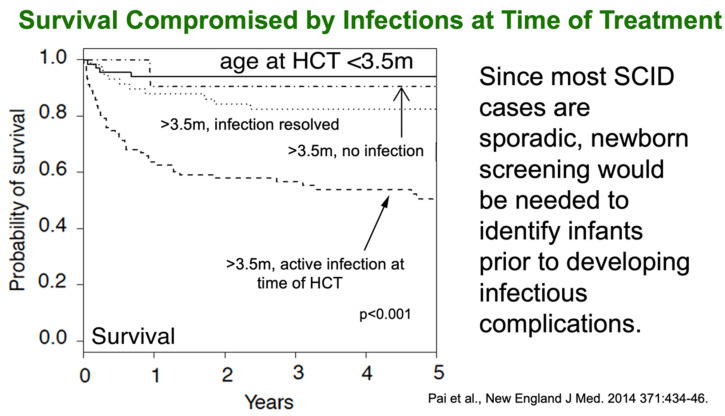
Survival compromised by infections at time of treatment [3]. HCT, hematopoietic cell transplantation.

As with Dr. Buckley’s data, the PIDTC data showed dramatic differences in survival according to age at transplant and infection history (Figure 3) [3]. The top solid line in the PDITC survival graph represents infants transplanted at less than 3.5 months of age, while the dotted lower line with only about 50% survival represents patients who were transplanted at over 3.5 months of age in the face of an active infection. 

The original criteria of Wilson and Jungner used to justify the inclusion of a disorder in population-based NBS are met by SCID, which is fatal in the first year of life if untreated [2]. Newborns with SCID appear healthy and are not readily detected without screening. While the incidence of SCID was unknown when the first pilot screening programs started, certain populations such as Navajo Native Americans have a high incidence due to founder mutations, and we now know the general population incidence in the USA is around 1 in 66,000 births [4]. Effective treatment is available using allogeneic hematopoietic cell transplantation, enzyme injections for ADA deficiency, or, increasingly, gene therapy. Finally, as shown above, earlier treatment leads to better survival. 

## 3. Screening Test to Detect SCID: T-Cell Receptor Excision Circles (TRECs)

**Figure 4:**
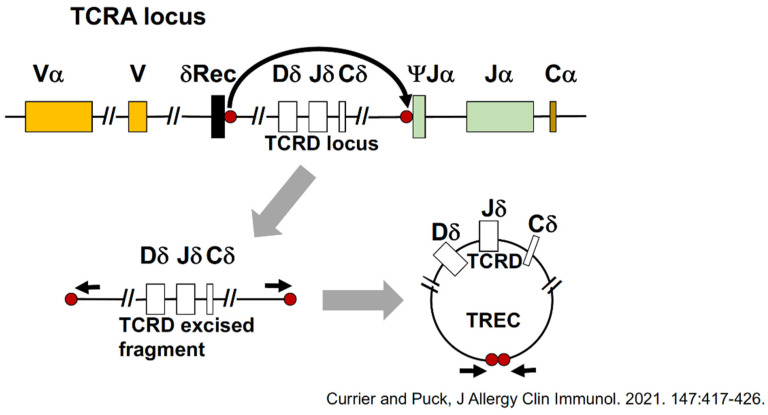
T-cell receptor excision circles (TRECs): a biomarker for normal T-cell development [7]. TCRA, T-cell receptor alpha; TCRD, T-cell receptor delta; V, variable; D, diversity; J, joining; C, constant regions of T-cell receptor genes; Rec, recombination sequence for TCRD.

Since the end of 2018, all states in the USA have had NBS for SCID and have found it cost-effective. The only element missing initially was an accurate, inexpensive, high-throughput screening test, preferably taking advantage of the dried blood spots (DBSs) already being collected to test newborns for metabolic and other diseases. Assaying DBS DNA for T-cell receptor excision recombination circles (TRECs) has proven to be a worthy test for this purpose [5,6]. Figure 4 below shows how the T-cell receptor alpha gene locus (TCRA) undergoes a recombination to remove the TCR delta locus (white boxes) from what will become the mature linear gene, and this excised delta fragment is made into a circle by the same enzymes that are joining the linear gene segments. Quantitative PCR amplifying the DNA, including the joined ends of the TREC circle, shown as red dots, reveals the number of TRECs in a DBS punch [7].

## 4. Outcomes of Newborn Screening Programs for SCID in the USA

**Figure 5:**
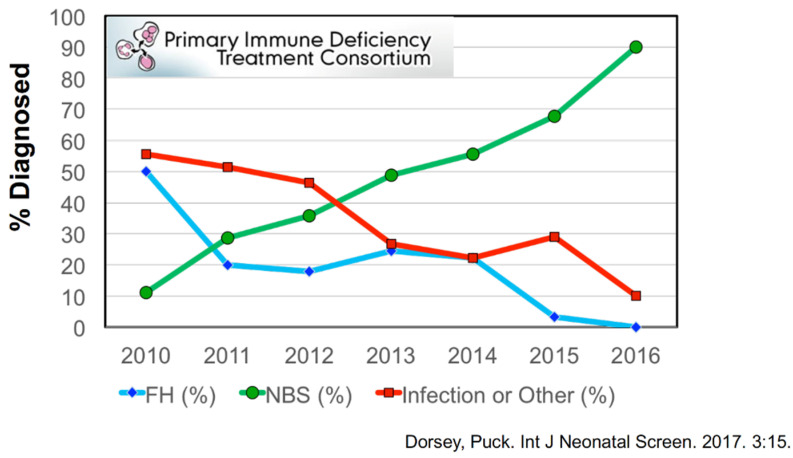
PIDTC percentage of SCID cases diagnosed by each presented finding: newborn screening (NBS) vs. infection or other clinical illness vs. family history (FH) [10].

TRECs have proven to be an excellent biomarker for the presence of sufficient newly formed T cells for effective immune responses [4,7,8]. When infants have SCID, their NBS TRECs are extremely low or undetectable, provided control DNA amplification is present. The first pilot screening program for SCID started in 2008 in Wisconsin and was followed by trials on the Navajo Indian Reservation [9], where SCID occurs in 1 in 2000 births; in Massachusetts, California; and in New York beginning in 2009–2010 [8]. As more individual state health departments in the USA added SCID screening with TRECs to their NBS panels, the diagnosis of SCID changed dramatically (Figure 5) so that, by 2016, 90% of new cases enrolled in the PIDTC studies had been detected by NBS (green), while the number presenting with infections substantially declined [10].

## 5. Experience with SCID Newborn Screening in California

**Figure 6:**
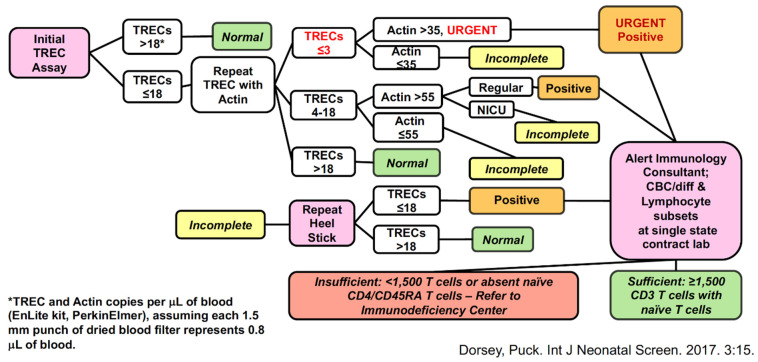
California SCID newborn screening TREC test and lymphocyte subsets [10].

The California SCID NBS algorithm is shown below (Figure 6) [10]. As diagrammed, a TREC PCR is done initially and, if the value is normal (greater than 18 TRECs/µL of blood, using the Perkin Elmer Enlite^®^ kit), no further action is taken. If TRECs are below this cutoff, further punches are tested for TREC and control actin gene PCR. If TRECs remain undetectable or at only 1–3/µL with adequate control PCR, extra urgency is required to recall infants for a liquid blood sample for complete blood count and lymphocyte subset determination, including naïve and memory CD4 and CD8 T-cell subsets. Other infants with abnormally low TRECs may have a repeat DBS test if they are preterm or in a neonatal intensive care unit (NICU), but otherwise they also are recalled for lymphocyte enumeration by flow cytometry. Unlike many other SCID NBS programs in the USA, California has included the follow-on lymphocyte subset determination as part of the NBS program, using a single laboratory to perform immune phenotyping. Designated immunology experts in each region of the state review and interpret the results. If the total T-cell count is greater than 1500/µL and naïve T lymphocytes are present in substantial proportions, the infant is excused from further follow-up. Those with significant T-cell lymphopenia are referred to immunology centers for further clinical and laboratory evaluation and management.

**Figure 7:**
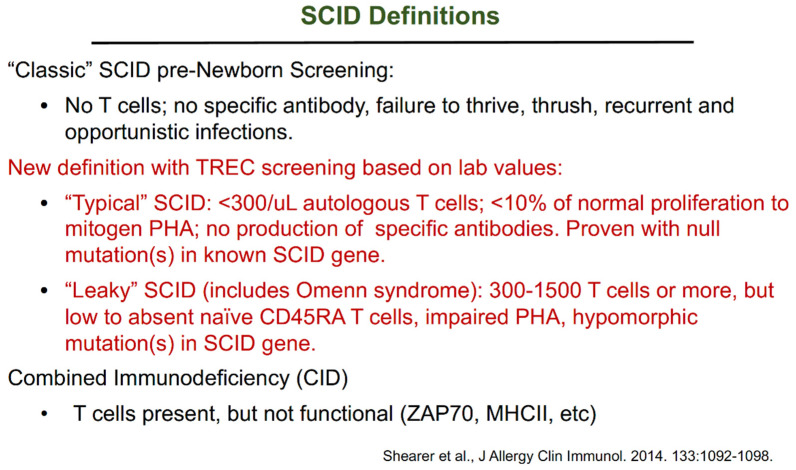
SCID definitions [11].

We have had to change the definitions of SCID from the classic picture based on infectious complications (Figure 7; [11]) because most infants with SCID diagnosed following NBS do not show failure to thrive, thrush, or recurrent infections. Rather, they appear to be healthy and thriving, requiring the updated SCID definition to be based on lab values as adopted by the PIDTC. 

It is important to note that combined immunodeficiency (CID) disorders may not be identified by the TREC test because T cells and TRECs are present but T cells are not functional in many cases; examples include ZAP70 deficiency or MHC class II deficiency. Moreover, hypomorphic mutations in SCID genes, known to result in delayed or late onset of disease, may rarely have normal TREC numbers at birth. In California, two cases out of 3.25 million births had normal TREC newborn screens but later developed recurrent infections and were diagnosed with late-onset SCID [4].

**Figure 8:**
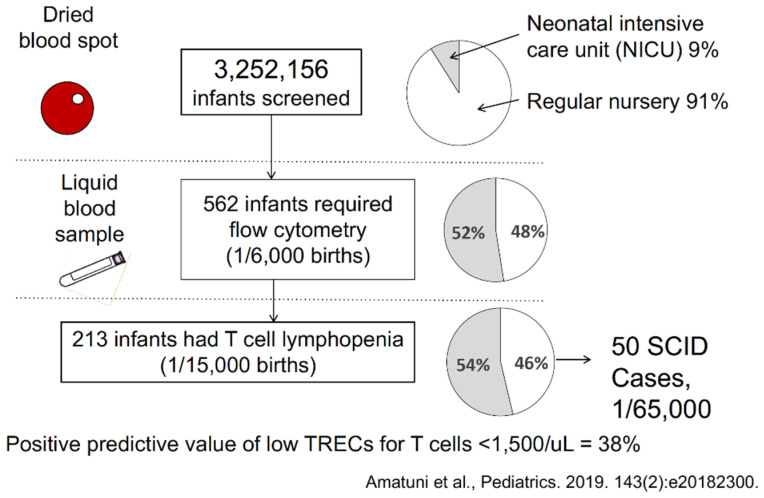
Diagram of numbers of cases at each stage in California SCID newborn screening during the first 6.5 years [4]. Left, infant samples tested; right, proportion of infants cared for in regular nurseries (white) vs. intensive care nurseries (gray).

The California SCID NBS experience over its first 6.5 years has been published, encompassing 3.25 million infants screened [4]. As diagrammed below (Figure 8), a larger proportion of NICU infants (gray pie segments) required follow-on flow cytometry testing than did infants in regular nurseries, but the total of all infants who required a liquid blood sample was only 562, or only 1 per 6000 births. Of those, only 213 had fewer than 1500 T cells/µL, making the positive predictive value of the TREC test for having low T cells very high for an NBS test at 38%.

**Figure 9:**
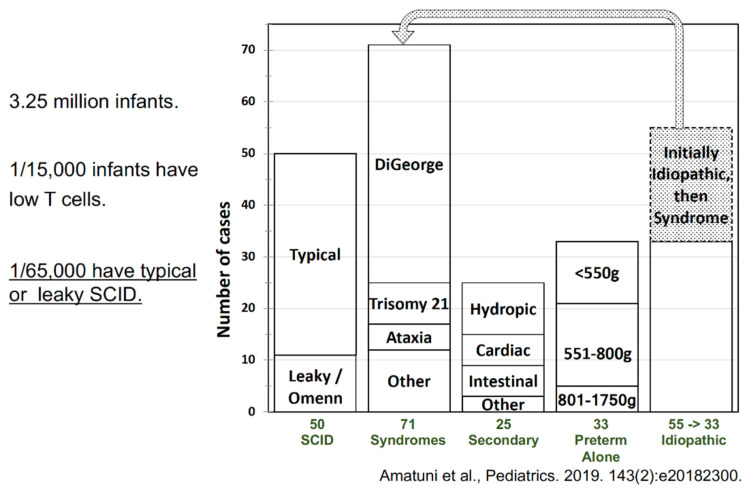
Final diagnoses of infants with abnormal TREC screens and low T cells who were identified by the California SCID NBS program over 6.5 years [4].

What was the ultimate diagnosis of the children who had positive SCID screens and low T cells by flow? The bar chart below (Figure 9) shows that there were 50 cases out of the 3.25 million infants screened who had SCID, including leaky SCID and Omenn syndrome (1 per 66,000) [4]. There were 71 infants ultimately diagnosed with syndromes, including 22q interstitial deletion DiGeorge syndrome, the most common by far; a substantial number of those initially considered idiopathic had a syndromic diagnosis established during the first several months of life. There were 25 infants with secondary defects, such as hydrops, congenital heart disease, and other congenital abnormalities associated with enhanced T-cell loss from the peripheral circulation, and some infants whose mothers had received immunosuppressive medications that crossed the placenta during pregnancy. These latter infants experienced T-cell recovery once the drug effects had ceased. Transiently low T cells were observed in a small proportion of pre-term, low-birthweight infants.

Finally, there was the category of idiopathic T-cell lymphopenia; initially, there were 55 infants in this category but, as shown in the chart, 22 of them were moved to the “Syndromes” category after diagnosis of DiGeorge syndrome or other conditions that were confirmed after the NBS had alerted physicians to look for them. Of the remaining infants with idiopathic T-cell lymphopenia, about one third improved over time, about one third continued to have low numbers of T cells, and one third eventually received a transplant [4].

**Figure 10:**
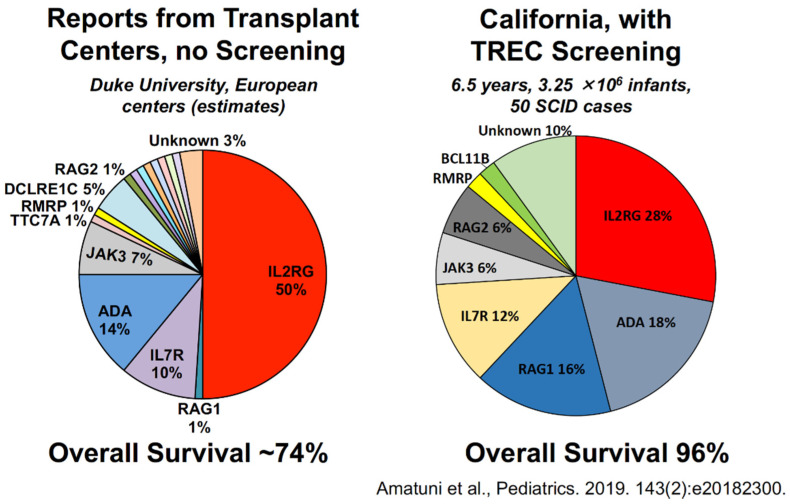
Genotypes of typical and leaky SCID [4].

The distribution of genotypes of SCID found by unbiased screening of the whole population differed from historical reports, as shown below (Figure 10) [4]. The left pie chart illustrates genotypes reported from transplant centers prior to the NBS era, in which about half of the cases had the X chromosome-linked *IL2RG* genotype; but IL2RG defects were observed in only about a quarter of SCID cases found by NBS in California. NBS in California also showed relatively more SCID cases due to *RAG1/2* deficiency and more cases with unknown genotypes.

SCID was found by NBS in California in about 1 per 66,000 births (96% confidence interval, 1 per 51,000 to 1 per 90,000) [4], making SCID less common than phenylketonuria but more common than galactosemia, two disorders for which NBS is well-established. The incidence of all T-cell lymphopenias requiring intervention (including isolation from infections, withholding of live rotavirus vaccinations, and, for some cases, prophylactic antibiotics, immune globulin infusions, or immune restoring transplants) was about 1 in 20,000. These numbers support the idea that SCID screening is cost-effective.

**Figure 11:**
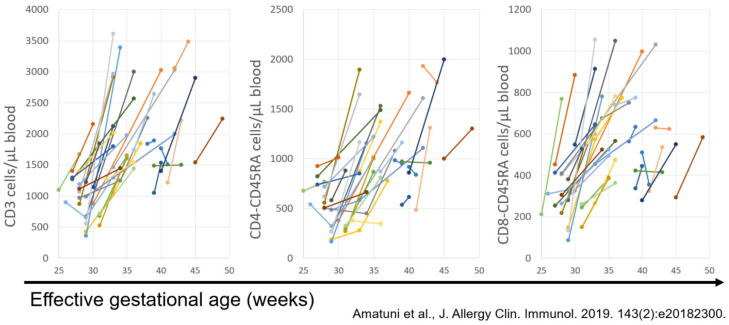
T cells and subsets from 33 preterm infants without immune defects, measured sequentially, show increases with age [12].

Finally, the California NBS program was able to establish new, accurate normal values for T-cell subsets, including naïve- and memory-phenotype helper and cytotoxic T-cell subsets, at gestational ages from 25 weeks to full term, which we published in 2019 [12]. This publication included serial sampling over time in 33 premature infants, documenting that their T-cell counts rose to normal levels as they approached full term, as shown below (Figure 11).

In summary, the extensive experience in the USA, and in particular in California, with population-based SCID NBS for SCID has proven that this screening is successful in identifying infants who truly have SCID and bringing them to early treatment. Moreover, the flagging of individuals with non-SCID T-cell lymphopenia is beneficial because it allows protective measures and other appropriate management to be instituted sufficiently early to avoid infectious complications. Finally, compared to NBS for other disorders, NBS for SCID has high specificity, so the family anxiety and expense associated with false positive cases is minimized. These existing programs have provided ample data to justify institution of SCID NBS in other countries.

## Figures and Tables

**Table 1:** Many SCID genes, distinct T, B, and NK cell profiles. The genes below are most commonly found to cause SCID.

Gene (Disease Name)	T Cell Profile	B Cell Profile	Natural Killer Cell Profile
*L2RG* (common γ chain, X-linked)	T−	B+	NK−
*JAK3* (γ chain-associated Janus kinase)	T−	B+	NK−
*IL7R* (IL-7 receptor α chain)	T−	B+	NK+
*CD45* (membrane tyrosine kinase)	T−	B+	NK+
*TCRD/E/Z* (TCR CD3 δ, ε, and ξ chains)	T−	B+	NK+
*RAG1/RAG2*	T−	B−	NK+
*DCLRE1C* (Artemis)	T−	B−	NK+
*LIG4* (DNA ligase IV)	T−	B−	NK+
*PRKDC* (DNA PKcs)	T−	B−	NK+
*ADA* (adenosine deaminase)	T−	B−	NK−
*AK2* (reticular dysgenesis, deafness)	T−	B+/−	NK+
*TTC7A* (multiple bowel atresias)	T−	B+/−	NK+
*RMRP* (cartilage hair hypoplasia)	T−	B+/−	NK+
*FOXN1* (nude mouse)	T−	B+	NK+
*CORO1A* (coronin 1A)	T−	B+/−	NK+

presence (+) or absence (−) of indicated subset.
